# Selenium and Arsenic Levels, Prevalence of Common Variants of Genes Involved in Their Metabolism, and Psoriasis Disease

**DOI:** 10.3390/biomedicines12051082

**Published:** 2024-05-13

**Authors:** Tadeusz Dębniak, Piotr Baszuk, Ewa Duchnik, Karolina Rowińska, Emilia Rogoża-Janiszewska, Magdalena Boer, Magdalena Kiedrowicz, Mariola Marchlewicz, Daniel Watola, Martyna Feherpataky, Róża Derkacz, Anna Dębniak, Wojciech Marciniak, Katarzyna Gołębiewska, Jan Lubiński, Rodney J. Scott, Jacek Gronwald

**Affiliations:** 1 Department of Genetics and Pathology, International Hereditary Cancer Center, Pomeranian Medical University, 71-252 Szczecin, Poland; 2 Department of Skin Diseases and Venerology, Pomeranian Medical University, 72-010 Police, Polandmariola.marchlewicz@pum.edu.pl (M.M.);; 3 Read-Gene, Grzepnica, 72-003 Dobra, Poland; 4 Medical Genetics, School of Biomedical Sciences and Pharmacy, Callaghan, NSW 2308, Australia; rodney.scott@newcastle.edu.au

**Keywords:** psoriasis, serum, selenium, arsenic

## Abstract

Using an Inductively Coupled Plasma Mass Spectrometer we measured the concentration of selenium and arsenic in serum and blood samples from 336 unselected psoriatic patients and 336 matched healthy controls to evaluate any associations with the clinical course of the disease. We genotyped 336 patients and 903 matched controls to evaluate the prevalence of *SOD2* (rs4880), *CAT* (rs1001179), *GPX1* (rs1050450), and *DMGDH* (rs921943) polymorphisms using Taqman assays. The mean selenium (Se) level in serum was 74 µg/L in patients and 86 µg/L in controls (*p* < 0.001). The mean Se level in blood was 95 µg/L in patients and 111 µg/L in controls (*p* < 0.001). Psoriasis risk was greatest among participants with the lowest serum (<68.75 µg/L, OR: 8.30; *p* < 0.001) and lowest blood concentrations of Se (<88.04 µg/L, OR: 10.3; *p* < 0.001). Similar results were observed in subgroups of males and females. We found an inverse correlation of selenium levels with PASI, NAPSI, and BSA scores. There was no significant difference in the distribution of the *CAT*, *GPX1*, *DMGDH*, and *SOD2* polymorphisms. Among carriers of rs4880, rs1001179, and rs921943 polymorphisms, blood selenium levels were significantly lower. The mean arsenic level in serum was 0.79 µg/L in patients and 0.7 µg/L in controls (*p* = 0.2). The mean concentration in blood was 1.1 µg/L in patients and 1.3 µg/L in controls (*p* < 0.001). In conclusion, we found that lower selenium levels, in blood and serum, are associated with psoriasis risk and its more severe course. Future prospective studies should focus on the optimalisation of the concentration of this trace element not only for prophylactic guidance but also to support the treatment of this disease.

## 1. Introduction

Psoriasis is a common skin disease which affects almost 3% of European population [1]. It is a recurrent autoimmunological disorder with the increased proliferation and accelerated maturation of keratinocytes due to the overexpression of many proinflammatory cytokines and increased reactive oxygen species (ROS) [2,3]. It’s pathogenesis is yet not known. Both genetic alterations and environmental factors, including obesity, diet, alcohol, or stress, play a significant role in the development of psoriasis. Microelements, such as selenium, zinc, and copper, are involved in the destruction of free radicals through cascading enzyme systems [4]. Arsenic is another trace element which affects apoptosis in keratinocytes [5]. It also influences inflammation related to antioxidant processes [6].

Recent studies on the role of microelements in the pathogenesis and treatment of psoriasis have shown controversial findings and are still limited. There are only a few reports in the literature about selenium concentrations in psoriasis and only one about arsenic (As) levels. The reports to date are based on small patient numbers and present inconsistent data, the majority of which are based only on serum concentrations of Se or As. Due to the paucity of reports in the literature, it is justified to undertake larger research-based studies of cases and controls. Herein, in order to more precisely determine the impact of selenium and arsenic levels on psoriasis occurrence and its clinical course, we measured the concentration of both microelements in the serum and blood from 336 unselected patients with psoriasis plus 336 healthy controls to evaluate any possible associations with the clinical course of the disease such as length and severity (measured by PASI, NAPSI, and BSA scales), treatment, family history, and the coincidence of cardiovascular, metabolism, or cancer diseases. The outcome of this study is necessary for future studies that focus on recurrence, prophylactics, and psoriasis treatment through the optimization of Se and As levels. Finally, the simultaneous evaluation of blood and serum levels of microelements in psoriatic patients is important from a practical perspective as it can lead to the optimization of routine microelement examination through the selection of full blood or serum samples.

The key role of selenium in human metabolism is attributed to its presence in glutathione peroxidase (GSH-Px), which protects cells against the harmful effects of free radicals, i.e., reactive oxygen species (ROS) and may induce apoptosis in keratinocytes [3,7,8]. Among antioxidant enzymes involved in oxidative stress, catalase (*CAT*), glutathione peroxidase 1 (*GPx1*) and superoxide dismutases (*SODs*) play key roles in the detoxification of ROS. Altered activities of these enzymes have previously been reported in psoriatic patients [9,10,11,12]. 

The *SOD2* gene codes the MnSOD protein, known to be a major superoxide detoxifying enzyme of cells. The *SOD2* rs4880 variant, described either as a missense mutation (Val16Ala) or a change in the regulatory 5′UTR sequence, has been reported to be associated with arthropathic psoriasis [13]. In a second study (based on the examination of a small study group), no significant difference in the prevalence of rs4880 was found between psoriatic patients and control subjects [14], highlighting the problems with small studies.

*CAT* encodes the enzyme catalase, which limits the deleterious effects of ROS. The rs1001179 variant (c.-262C > T) is located in the promoter region of *CAT*, where it influences transcription factor binding and alters the basal transcription rate and the consequent expression of the encoded enzyme [15]. It has not been studied in psoriasis until now. 

*GPX1* codes for the most abundant isoenzyme of the four different GPxs identified to date; it is also a selenium-dependent enzyme that is ubiquitously expressed and protects against oxidative damage by reducing hydrogen peroxide and a wide range of organic peroxides with reduced glutathione. The only study of the *GPX1* rs1050450 variant in psoriasis, described as missense Pro200Leu variant, suggested a lack of association between the variant and psoriasis occurrence [14], but the study was based on a small group of patients and this result awaits confirmation. 

*DMGDH* encodes a protein involved in the metabolism of analogous selenium compounds such as selenomethionine. Two recently published studies showed that concentrations of both whole-blood and toenail selenium were significantly associated with the *DMGDH* rs921943 genotype. The *DMGDH* rs921943 genotype appears to affect baseline (steady-state) selenium status [16,17]. The variant has not been studied in psoriasis thus far.

Association studies of DNA alterations in genes suspected to be involved in the pathogenesis of this disease can lead to the identification of inherited risk factors of psoriasis, which is crucial for future studies focused on individualized therapy depending on the molecular status of the cases.

The main aim of our study is the evaluation of the levels of selected microelements involved in antioxidant processes and inflammation in the serum and blood of patients with psoriasis compared to healthy controls. The second objective is to determine the prevalence of selected polymorphisms in genes coding the proteins crucial for the metabolism of these microelements. 

The ultimate goal is to evaluate possible associations between the serum/blood levels of the microelements, the presence of analyzed DNA recurrent mutations/polymorphisms, and the occurrence/course of psoriasis disease.

## 2. Materials and Methods

### 2.1. Patients

In the first stage of the study, a group of 336 randomly selected adult patients with skin psoriasis was established. The consecutive cases were recruited from the Dermatology and Venerology Clinics of the Pomeranian Medical University (PMU) in Szczecin and outpatients departments in Szczecin. Participation rate exceeded 76%. The inclusion criteria were the diagnosis of skin psoriasis confirmed by dermatologist and an age of 18 or more. There were 209 males (62%) and 127 females (38%) among psoriasis patients. 

Clinical and pedigree data were collected (including year of birth, treatment, BSA, PASI and NAPSI scales, the coincidence of other diseases such as cardiovascular, metabolic, or malignancies, family history, and smoking) (Table 1).

### 2.2. Controls

A total of 336 sex- and age-matched (+/− 2 years) healthy adults were used as control subjects for selenium and arsenic level assessment. For the evaluation of the prevalence of the four common DNA polymorphisms found in *SOD2*, *CAT*, *GPX1*, and *DMGDH*, respectively, 903 sex- and age-matched (+/− 2 years) healthy adults (324 females and 579 males) were enrolled (3:1 control–case match ratio; for 35 cases we did not manage to fully match controls). The control group was collected from the registry located at the Department of Genetics and Pathology of the Pomeranian Medical University. 

All study subjects provided a signed consent form for participation in the study. During the interview, the goals of the study was explained, informed consent obtained, genetic counseling given, and a blood sample taken for DNA, blood, and serum Se and As analyses.

### 2.3. Methods

Venous blood samples (40 mL) of all patients and controls were obtained in the morning (fasting) and frozen at −80 °C within 2 h following collection. The samples were sent within 2 days to the Department of Genetics and Pathology where DNA was isolated from 5 mL of peripheral blood using standard methods. The remaining material was used for the evaluation of the concentration of microelements [18].

DNA was genotyped for the detection of the rs4880 variant in *SOD2*, rs1001179 in *CAT*, rs1050450 in *GPX1*, and rs921943 in *DMGDH* using a TaqMan assay (Applied Biosystems/Life Technologies, Carlsbad, CA, USA) and the LightCycler Real-Time PCR 480 system (Roche Diagnostics GmbH, Basel, Switzerland). The primer and probe sequences as well as PCR conditions are available upon request “https://www.thermofisher.com/pl/en/home.html accessed on 7 May 2024)”. Laboratory technicians were blinded to case–control status. The overall genotyping call rate was 99.3%. Randomly selected samples were verified using Sanger sequencing.

Blood and serum concentrations of selenium and arsenic were analyzed in 300 cases and 300 matched controls using an Inductively Coupled Plasma Mass Spectrometer (ICPMS, PerkinElmer, Waltham, MA, USA) [19]. Calibration standards were prepared by means of the dilution of 10 mg/L Multi-Element Calibration Standard 3 (PerkinElmer Pure Plus, PerkinElmer Life and Analytical Sciences, Waltham, MA, USA) with reagent blank consisting of 0.65% solution of nitric acid (Merck, Darmstadt, Germany) and 0.002% Triton X-100 (PerkinElmer, USA). Calibration curves were created using four different concentrations: 0.1 µg/L, 0.5 µg/L, 1 µg/L, and 2 µg/L. Germanium (PerkinElmer Pure, PerkinElmer Life and Analytical Sciences, USA) was used as an internal standard, and ClinChek^®^ Plasma Control Level I (Recipe, Munich, Germany) was used as a reference material. The reference material was measured after each of the six samples. If the difference in the reference material measurements was greater than 5%, the entire series was repeated. Each sample was measured in duplicate from different analytical runs. Prior to analysis, all samples were centrifuged (4000× *g*, 15 min) and the supernatant diluted 100 times with the reagent blank. Technical details, plasma operating settings, and mass spectrometer acquisition parameters are available on request.

### 2.4. Statistical Analyses

Selenium and arsenic levels were assigned into one of four quarters (Q1–Q4) determined on each element’s values, arranged in ascending order. In all cases, the reference quarter was the range in which the number of diagnosed cases of psoriasis was the lowest. In order to estimate the potential association between selenium and arsenic (in blood and serum samples), univariable logistic regression models were calculated based on all enrolled subjects and on subgroups of females and males separately (logistic regression models were not adjusted for confounding variables).

In order to determine differences in selenium and arsenic blood levels among variants of individual genes, nonparametric Wilcoxon rank-sum tests were performed. In addition, variants of individual genes (*SOD2*, *CAT*, *GPX1*, and *DMGDH*) were analyzed in groups that consisted of 336 psoriasis patients and 903 matched controls for potential association between their presence and the occurrence of psoriasis. The odds ratios (OR), 95% confidence intervals (CI), and test probability values (*p*) were also estimated using univariable logistic regression models.

The logistic regression models were also used in order to verify the potential interactions between selenium blood levels and each of the individual gene variants. In parallel, the associations between psoriasis occurrence and selenium/arsenic blood levels in subgroups composed of carriers of variants (CC/nonCC) of individual genes were tested. In order to estimate the presence of collinearity between quantitative variables among psoriasis patients, Spearman’s rank correlation coefficients (ρ) were calculated.

In all statistical procedures, the test probability values below 5% were considered as statistically significant. All calculations were performed using R statistical environment (R: A Language and Environment for statistical Computing, Vienna, Austria 2023).

## 3. Results

The mean serum selenium levels were 86 µg/L and 73.89 µg/L for controls and psoriasis patients, respectively (*p* < 0.001). The blood selenium level was also lower in psoriasis patients (94.88 µg/L), compared to the control group (111.18 µg/L) (*p* < 0.001). Psoriasis occurrence among study participants with a low serum selenium level (Q1: 25.93 µg/L–68.75 µg/L) was over eight times higher (OR: 8.30; 95% CI: 4.55–15.1; *p* < 0.001), compared to the baseline selenium quarter (Q4: 88.98 µg/L–214.02 µg/L). This association was even stronger (OR: 10.3; 95% CI: 5.09–20.8; *p* < 0.001) for participants with a low blood selenium level (Q1: 36.15 µg/L–88.04 µg/L), compared to the highest quarter (Q4: 111.78 µg/L–306.34 µg/L) of concentrations (Table 2).

Similar results to those obtained on the entire study group analysis were observed in subgroups of males and females. For both subgroups, low serum and blood selenium levels (Q1) were associated with increased psoriasis occurrence (Table 3).

The mean serum arsenic level was 0.7 µg/L and 0.79 µg/L (*p* = 0.2) in controls and patients, respectively; however, the mean blood arsenic concentration was higher in controls (1.3 µg/L) than in patients (1.09 µg/L) (*p* < 0.001). Among study participants with high serum arsenic levels, psoriasis was more frequent (OR: 2.27; *p* < 0.001, OR: 2.06; *p* = 0.003 and OR: 1.61; *p* = 0.048 for Q2–Q3, respectively) compared to study participants with low serum levels (Q1: 0.17 µg/L–0.32 µg/L). Surprisingly, the opposite association was observed for blood arsenic levels, where psoriasis was more frequent (OR: 3.82; 95% CI: 2.08–7.04; *p* < 0.001) among patients with the lowest quarter of blood arsenic levels (Q1: 0.27 µg/L–21.68 µg/L), compared to the reference quarter (Q4) (Table 2).

We found no significant differences in the distribution of *SOD2* (rs4880), *CAT* (rs1001179), *GPX1* (rs1050450), or *DMGDH* (rs921943) among the 336 cases and 903 healthy controls (Table 4).

Among carriers of the rs4880 variant in *SOD2,* rs1001179 in *CAT*, and rs921943 in *DMGDH*, the blood selenium levels were significantly lower (Table 5).

No statistically significant interactions were observed between selenium blood levels and each of the individual genes and psoriasis frequency (*p* = 0.7; *p* = 0.085; *p* = 0.07; and *p* = 0.07) for *SOD2*, *CAT*, *GPX1*, and *DMGDH*, respectively.

The evaluation of clinical data revealed that there were significant, negative correlations between blood selenium levels and PASI (Spearmans ρ = −0.39) and NAPSI (ρ = −0.09) scores. Significantly lower blood selenium levels were observed among psoriasis patients with BSA score ≥10 (*p* < 0.001), liver disorders (*p* = 0.041), and smokers (*p* < 0.001).

Blood selenium levels were also positively correlated with LDL (ρ = 0.26) and were significantly higher among psoriasis patients with hypercholesterolemia (*p* = 0.014) and those with gluten intolerance (*p* = 0.026).

There were no significant differences in blood selenium levels among psoriasis patients in relation to cardiovascular diseases (*p* = 0.5), diabetes (*p* = 0.8), Hashimoto disease (*p* = 0.072), lactose intolerance (*p* = 0.5), malignancy treatment (*p* > 0.9), PUVA/UVB treatment during the previous 6 months (*p* = 0.6), or alcohol consumption (*p* = 0.11).

## 4. Discussion

There is a paucity in the literature about possible associations between selenium and psoriasis and what does exist is limited and inconsistent.

Decreased serum levels of selenium in psoriasis were observed by Kadry et al., who examined 30 patients [20], Wacewicz et al., studying 60 patients [21], and Serwin et al., who assessed 30 patients [20]. Similarly decreased selenium concentrations in serum and also in blood taken from psoriatic cases were reported by Seneczko et al. [22] and Fairis et al. (serum and blood) [23].

No difference in selenium concentration between psoriatic patients and healthy controls was found by Tossi et al. (40 cases and 40 controls) [24] and Donadini et al. (64 cases) [25]. Increased levels of selenium were, however, observed in the study conducted by Elhaddad et al. [26].

Herein, we found that both serum and blood selenium levels were lower among patients when compared to healthy controls. Similar results to those obtained on the entire study group analysis were observed for subgroups of both males and females. The psoriasis risk among study participants was over 8 times greater in cases with the lowest serum selenium levels (<68.75 µg/L) and over 10 times greater in cases with the lowest blood selenium concentrations (<88.04 µg/L). Since we found very consistent results of both serum and blood selenium levels and due to the fact that blood measurements are easier to conduct in terms of logistics, blood can be used in order to create the best algorithm of evaluation of the selenium concentration in patients with psoriasis.

We were unable to demonstrate any link between selenium levels and the occurrence of other common disorders in our cases, such as cardiovascular or metabolic diseases, BMI, hypertension, previous treatment, the consumption of alcohol, or smoking status. We were also unable to demonstrate an association between selenium and Hashimoto disease onset in our patients with psoriasis (*p* = 0.072). The evaluation of the clinical data from psoriasis patients revealed a significant, negative correlation of blood selenium levels with PASI (ρ = −0.39) and NAPSI (ρ = −0.09) scores. The blood selenium levels of patients with BSA ≥10 were significantly lower (*p* < 0.001).

These results suggest that selenium can also impact the disease course. Thus, future prospective studies should focus on the optimalisation of the concentration of this trace element for prophylactic treatment to reduce psoriasis frequency and also to support the treatment of this disease.

Only three studies have evaluated the consequences of selenium supplementation in psoriasis. Fifty eight patients with psoriasis participated in a placebo-controlled clinical trial of selenium and Vitamin E supplementation. The investigators reported a significant improvement of psoriasis in a cohort receiving selenium [27]. In another study of 37 psoriatic cases, selenium supplementation combined with narrowband UVB therapy was compared with placebo plus phototherapy. The authors found no significant clinical differences between the two cohorts [28]. Finally, a small prospective trial of seven participants receiving selenium for a short period of six weeks revealed no change in the course of the disease. [29]. In the only published study on As, it was revealed that increased levels of arsenic were identified among patients with moderate and severe psoriasis [30]. Herein, we found that the mean serum arsenic levels were similar among cases and controls (0.8 µg/L vs. 0.7 µg/L), whereas the mean blood arsenic level was slightly higher in controls (1.3 µg/L vs. 1.1 µg/L). The psoriasis risk associated with As appears complex and quite different to that associated with Se. Intriguingly, serum As was higher among participants but low when assessed in whole blood. Additional studies are required to understand the differences between plasma and whole blood As levels and how they potentially interact in psoriasis.

Herein, we also examined the prevalence of four common polymorphisms within *SOD2* (rs4880), *CAT* (rs1001179), *GPX1* (rs1050450), and *DMGDH* (rs921943). No significant differences in their distribution among 336 cases and 903 controls were observed, suggesting that they are not linked to psoriasis and/or Se or As levels. The only result that was of note was the observation that blood selenium levels were significantly lower for carriers of the CC variant of *SOD2*, *CAT*, and *DMGDH*, which may be a generalized effect of these polymorphisms and the association between Se and psoriasis.

## 5. Conclusions

We found that lower selenium levels, both in blood and serum, are significantly associated with psoriasis risk and its more severe course. We observed that the selenium levels were significantly lower for carriers of the CC variant of the *SOD2*, *CAT*, and *DMGDH* genes.

The evaluation of possible associations between the examined microelements and psoriasis is an important step to study the significance of the optimalisation of the concentration of these microelements for prophylactics and to support the treatment of this disease. Another finding of this study is that both full blood and serum can be used in order to create the best algorithm of evaluation of the concentration of the examined microelements in patients with psoriasis.

Future prospective studies should focus on the optimalisation of selenium concentration not only for prophylactic guidance but also to support the treatment of this disease.

## Figures and Tables

**Table 1:** Clinical characteristics of psoriasis patients.

Variables	Psoriasis Patients, n = 336	Unknown
**Sex**		
Females	127 (38%)	
Males	209 (62%)	
**Psoriatic Arthritis**		48
No	204 (71%)	
Yes	84 (29%)	
**Diabetes**		50
No	262 (92%)	
Yes	24 (8.4%)	
**Cardiovascular Diseases**		52
No	224 (79%)	
Yes	60 (21%)	
**Liver Diseases**		53
No	270 (95.4%)	
Yes	13 (4.6%)	
**Malignancies**		53
No	270 (95%)	
Yes	13 (4.6%)	
**Smoking**		49
No	184 (64%)	
Yes	103 (36%)	
**Alcohol Consumption**		55
No	153 (54%)	
Yes	128 (46%)	
**Lactose Intolerance**		93
No	229 (94%)	
Yes	14 (5.8%)	
**Gluten Intolerance**		93
No	232 (95%)	
Yes	11 (4.5%)	
**Hashimoto Disease**		94
No	228 (94%)	
Yes	14 (5.8%)	
**Cholesterol**	86.45–747.20 (191.76)	77
**HDL**	23.60–103.99 (54.23)	83
**LDL**	26.09–230.53 (123.67)	82
**Glucose**	55.60–210.60 (99.17)	73
**PASI**		19
0–11	167 (53%)	
>=12	150 (47%)	
**BSA**		35
0–9	102 (35%)	
>=10	199 (65%)	
**BMI**	17.71–45.58 (28.07)	88
**PUVA/UVB during past 6 months**		119
No	213 (98%)	
Yes	4 (1.8%)	

n (%); min–max (mean).

**Table 2:** Psoriasis occurrence depending on selenium and arsenic blood/serum levels.

Frequency			Logistic Regression		
Variables	Controls n = 336	Psoriasis Patients n = 336	OR	95% CI	*p*
Serum selenium level	41.39–214.02 (86.00)	25.93–131.09 (73.89)			
Q1 25.93–68.75 (59.36)	38 (13%)	120 (36%)	8.30	4.55–15.1	<0.001
Q2 68.75–79.30 (74.26)	65 (22%)	92 (28%)	3.42	1.99–5.86	<0.001
Q3 79.33–88.92 (83.99)	92 (31%)	66 (20%)	1.50	0.89–2.54	0.13
Q4 (reference) 88.98–214.02 (101.03)	106 (35%)	52 (16%)	—	—	
Unknown	0 + 35	6			
Blood selenium level	54.63–306.34 (111.18)	36.15–190.36 (94.88)			
Q1 36.15–88.04 (76.77)	23 (8.8%)	124 (38%)	10.3	5.09–20.8	<0.001
Q2 88.05–99.80 (94.59)	65 (25%)	82 (25%)	2.03	1.16–3.54	0.013
Q3 99.81–111.68 (105.57)	79 (30%)	68 (21%)	1.54	0.91–2.62	0.11
Q4 (reference) 111.78–306.34 (131.63)	95 (36%)	52 (16%)	—	—	
Unknown	39 + 35	10			
Serum arsenic level	0.20–16.36 (0.70)	0.17–8.92 (0.79)			
Q1 (reference) 0.17–0.32 (0.28)	96 (32%)	62 (19%)	—	—	
Q2 0.32–0.42 (0.37)	59 (20%)	98 (30%)	2.27	1.43–3.61	<0.001
Q3 0.42–0.72 (0.54)	68 (23%)	89 (27%)	2.06	1.27–3.32	0.003
Q4 0.73–16.36 (1.78)	78 (26%)	81 (25%)	1.61	1.00–2.59	0.048
Unknown	0 + 35	6			
Blood arsenic level	0.29–21.68 (1.30)	0.27–12.17 (1.09)			
Q1 0.27–0.51 (0.42)	34 (13%)	113 (35%)	3.82	2.08–7.04	<0.001
Q2 0.52–0.79 (0.65)	73 (28%)	74 (23%)	1.25	0.74–2.11	0.4
Q3 0.79–1.16 (0.94)	77 (29%)	70 (21%)	1.14	0.67–1.92	0.6
Q4 (reference) 1.17–21.68 (2.72)	78 (30%)	69 (21%)	—	—	
Unknown	39 + 35	10			

n (%); min–max (mean).

**Table 3:** Psoriasis occurrence depending on selenium and arsenic blood/serum levels among males and females.

	Males			Females		
Variables	OR	95% CI	*p*	OR	95% CI	*p*
Serum selenium level						
Q1	8.58	3.99–18.5	<0.001	7.11	2.61–19.3	<0.001
Q2	3.63	1.88–7.00	<0.001	2.97	1.13–7.81	0.028
Q3	1.30	0.67–2.49	0.4	1.75	0.69–4.41	0.2
Q4 (reference)	—	—		—	—	
Blood selenium level						
Q1	15.3	5.46–42.7	<0.001	6.45	2.37–17.6	<0.001
Q2	1.69	0.83–3.45	0.15	2.54	1.01–6.39	0.047
Q3	1.61	0.83–3.12	0.2	1.55	0.64–3.78	0.3
Q4 (reference)	—	—		—	—	

**Table 4:** Allele distribution among cases and controls.

Gene	Controls, n = 903	Psoriasis Patients, n = 336	*p*
*SOD2*			
CC	243 (27%)	76 (25%)	
nonCC	660 (73%)	224 (75%)	0.6
Unknown	0	36	
*CAT*			
CC	545 (60%)	197 (64%)	
nonCC	358 (40%)	112 (36%)	0.3
Unknown	0	27	
*GPX1*			
CC	432 (48%)	152 (49%)	
nonCC	471 (52%)	157 (51%)	0.6
Unknown	0	27	
*DMGDH*			
CC	423 (47%)	144 (47%)	
nonCC	480 (53%)	165 (53%)	0.9
Unknown	0	27	

**Table 5:** Selenium blood levels and *SOD2*, *CAT*, *GPX1*, and *DMGDH* variants.

*SOD2*				*CAT*			*GPX1*			*DMGDH*		
Variable	CC	nonCC	*p*	CC	nonCC	*p*	CC	nonCC	*p*	CC	nonCC	*p*
Blood selenium level	36.15–219.42 (103.31)	56.92–306.34 (106.40)	0.025	36.15–261.86 (104.36)	46.65–306.34 (107.51)	0.034	36.15–272.06 (105.45)	46.65–306.34 (105.67)	0.6	36.15–306.34 (104.24)	46.65–261.86 (106.69)	0.023

n (%); min–max (mean).

## Data Availability

The datasets used and/or analyzed during the current study are available from the corresponding author on reasonable request.

## Abbreviations

SOD2	Superoxide Dismutase 2
CAT	Catalase
GPX1	Glutathione Peroxidase 1
DMGDH	Dimethylglycine Dehydrogenase
PASI	Psoriasis Area And Severity Index
NAPSI	Nail Psoriasis Severity Index
BSA	Body Surface Area
Se	Selenium
As	Arsenic
HDL	High Density Lipoprotein
LDL	Low Density Lipoprotein
PUVA	Psoralen Ultra-Violet A
UVB	Ultraviolet Radiation Burn
BMI	Body Mass Index
